# The evolving role of reirradiation in the management of recurrent brain tumors

**DOI:** 10.1007/s11060-023-04407-2

**Published:** 2023-08-25

**Authors:** Raffaella De Pietro, Lucy Zaccaro, Francesco Marampon, Paolo Tini, Francesca De Felice, Giuseppe Minniti

**Affiliations:** 1 Department of Radiological Sciences, Oncology and Anatomical Pathology, Sapienza University of Rome, Policlinico Umberto I, Rome, Italy; 2 Department of Medicine, Surgery and Neurosciences, University of Siena, Siena, Italy; 3 IRCCS Neuromed, Pozzilli (IS), Isernia, Italy

**Keywords:** Brain tumors, Recurrent glioblastoma, Recurrent ependymoma, Recurrent brain metastases, Reirradiation

## Abstract

Despite aggressive management consisting of surgery, radiation therapy (RT), and systemic therapy given alone or in combination, a significant proportion of patients with brain tumors will experience tumor recurrence. For these patients, no standard of care exists and management of either primary or metastatic recurrent tumors remains challenging.

Advances in imaging and RT technology have enabled more precise tumor localization and dose delivery, leading to a reduction in the volume of health brain tissue exposed to high radiation doses. Radiation techniques have evolved from three-dimensional (3-D) conformal RT to the development of sophisticated techniques, including intensity modulated radiation therapy (IMRT), volumetric arc therapy (VMAT), and stereotactic techniques, either stereotactic radiosurgery (SRS) or stereotactic radiotherapy (SRT). Several studies have suggested that a second course of RT is a feasible treatment option in patients with a recurrent tumor; however, survival benefit and treatment related toxicity of reirradiation, given alone or in combination with other focal or systemic therapies, remain a controversial issue.

We provide a critical overview of the current clinical status and technical challenges of reirradiation in patients with both recurrent primary brain tumors, such as gliomas, ependymomas, medulloblastomas, and meningiomas, and brain metastases. Relevant clinical questions such as the appropriate radiation technique and patient selection, the optimal radiation dose and fractionation, tolerance of the brain to a second course of RT, and the risk of adverse radiation effects have been critically discussed.

**Graphical Abstract:**

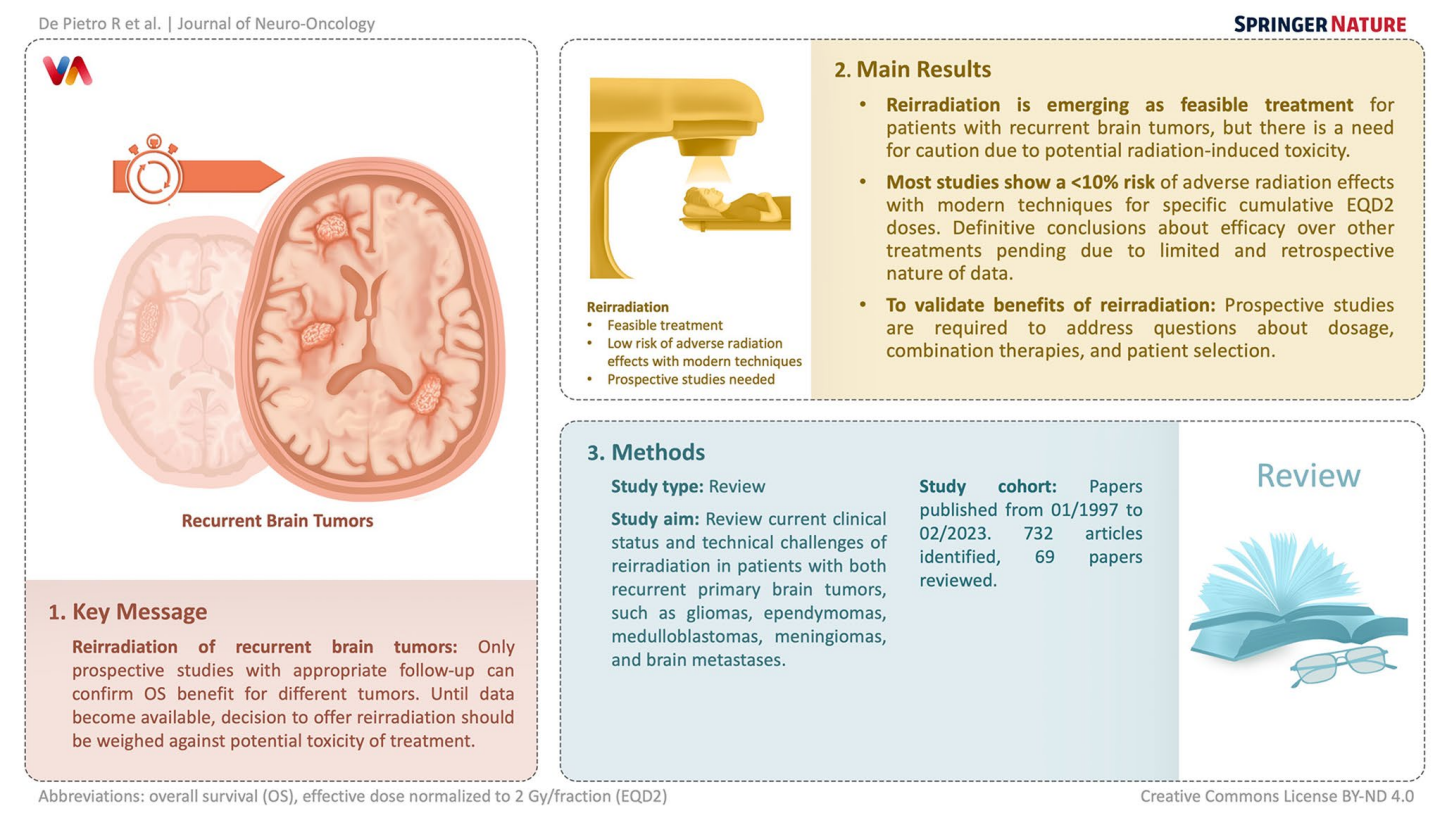

## Introduction

Radiation therapy (RT) remains an integral part of the management of most tumors of the central nervous system (CNS) [1]. Techniques have significantly improved in the last years resulting in new approaches to deliver highly conformal RT to the tumor while limiting the radiation dose to surrounding health brain tissues and organs at risk (OARs) [2]. Stereotactic radiotherapy (SRT), given as single-fraction stereotactic radiosurgery (SRS) or fractionated SRT, is frequently used for the treatment of small to medium size lesions due to its superiority in terms of dose conformity and rapid dose fall off outside the target compared with three-dimensional (3-D) conformal RT. Advanced techniques such as intensity-modulated RT (IMRT) and volumetric modulated arc therapy (VMAT) optimize the delivery of irradiation to large and irregularly shaped volumes. Finally, the use of appropriate image guided RT (IGRT) systems, using either orthogonal x-rays or cone beam computed tomography (CBCT), is necessary to reduce set-up margins and achieve high-precise patient positioning during treatment. Currently, there is a renewed interest in particle therapy using protons or heavier ions, because of their intrinsic physical and biological properties that consent to maintain highly conformal target coverage while sparing normal surrounding tissues and reducing the integral dose to the patient compared to most modern photon techniques [3].

In case of tumor relapse or progression, treatment options include surgery, chemotherapy or reirradiation, alone or in combination, as potentially salvage strategies. Due to its complexity, all treatment decisions require a multidisciplinary approach and should consider patients specific characteristics. Historically, radiation oncologists have been cautious regarding a second course of RT for tumors that have recurred in or close to the original treatment volume because of concerns about the risks of adverse radiation effects, such as radiation-induced brain necrosis. However, evidence from preclinical animal models and results from clinical series shows that brain and spinal cord have marked repair potential suggesting that reirradiation may represent a feasible option in selected patients [4–8]. When performing reirradiation, it is essential to keep dose as low as possible to normal brain tissue surrounding the recurrent tumor and sensitive structures such as brain stem, spinal cord, and optic apparatus. With this purpose, high-precision stereotactic techniques that allow for highly accurate patient positioning and dose delivery have replaced conventional RT in clinical practice for the treatment of patients with recurrent tumors who are deemed to receive reirradiation [2].

We performed an overview of the available literature on reirradiation of different types of primary brain tumors, such as gliomas, ependymomas, medulloblastomas and meningiomas, and brain metastases. Radiobiological principles behind reirradiation and current clinical evidence on the efficacy and toxicity of reirradiation have been critically discussed.

## Methods

We conducted a literature search of the relevant data on reirradiation of brain tumors using PubMed and Scopus databases, in accordance with the Preferred Reporting Items for Systematic Reviews and Meta-Analyses (PRISMA) guidelines. The following combination of keywords were searched: “reirradiation”, “recurrent brain tumors”, “glioblastoma”, “ependymoma”, “medulloblastoma”, “brain metastases”, “diffuse intrinsic pontine glioma” and “meningioma”. Search was limited to papers published in English language from January 1997 to February 2023. Clinical trials, original research, review articles, case report and case series were included, and reference lists were carefully explored for relevant papers that would have been missed by electronic search. Based on the initial searches, a total of 732 articles were identified. After abstract screening, 593 articles were excluded (duplicates or irrelevant) and 139 articles were considered for full text review. Studies assessing the role of RT in recurrent CNS tumors but without details on clinical outcomes and/or reirradiation were excluded (n = 70). A total number of 69 papers were finally reviewed based on relevance to the scope of this review. Data on overall survival (OS), progression-free survival (PFS), and toxicity after photon or particle reirradiation were extracted and grouped according to the clinical scenario investigated.

### Brain tolerance to reirradiation and dose constraints

Normal brain tissue dose tolerance is the limiting factor when giving reirradiation. Data on tolerance and recovery of CNS structures come from both preclinical and clinical studies [4–8]. Experimental data from studies investigating spinal cord tolerance to irradiation indicate that the CNS has a capacity to recover from occult radiation injury following treatment [4, 5]. In pigs treated with prior RT at a total dose of 30 Gy given in 10 fractions of 3 Gy each, Medin et al. [5] showed that spinal SRS reirradiation performed after one year was not significantly associated with an increased risk of motor deficits compared to controls treated by SRS only [5]. In another series of 56 rhesus monkeys assigned to receive two radiation courses to the cervical and upper thoracic spinal cord, 44 Gy during the first course and 57.2 Gy at the time of reirradiation given in 2.2 Gy per fraction, Ang et al. [4] reported a substantial recovery of occult injury within the first year following the initial course of RT. An additional recovery up to 100% was seen between 1 and 3 years, with no evidence of myelopathy for cumulative dose less than 110 Gy. Based on these experimental data, the authors suggested to use an estimated recovery of occult injury after a first course of RT around 50% at 1 year, 60% at 2 years, and 65–70% at 3 years or more, for the reirradiation of selected patients. Overall, for reirradiation of the full cord cross-section at 2 Gy per day after prior conventionally fractionated treatment, cord tolerance appears to increase at least 25% six months after the initial course of RT [6–8]. Although most data refer to spinal cord tolerance, the pathogenesis of brain radiation toxicity and its potential recovery is assumed to be like the one in spinal cord because of their low α/β ratio.

An estimated risk of symptomatic brain necrosis has been determined in patients with brain tumors following both SRS and SRT [6, 9–11]. A risk around 5% can be predicted following biologically equivalent dose (BED) of 72 Gy (range, 60–84 Gy) and 90 Gy (range, 84–102 Gy) with standard fractionation (1.8 to 2.0 Gy per fraction). For single-fraction SRS, volumes of normal brain receiving 12 Gy (V_12Gy_) of 5 cc, 10 cc, or > 15 cc have been associated with a risk of symptomatic radionecrosis around 10%, 15%, and 20%, respectively [10, 11]. Thus, the quantitative analysis of normal tissue effects in the clinic (QUANTEC) recommends limiting single-fraction V_12Gy_ to ≤ 5–10 cc (7). Based on dose/volume data and clinical risk estimates, maximum doses exceeding 55 Gy and 54 Gy in 1.8-2 Gy fractions for optic apparatus and brainstem, respectively, should be avoided in current clinical practice [12–14]. Following SRS, a maximum dose of 12 Gy to brainstem and 8 Gy to the optic apparatus is recommended [12, 14, 15]; of note, the risk of optic neuropathy remains low for maximum point doses of 10 to 12 Gy to small portions of the optic apparatus [16–19]. Data on tolerance doses of CNS organs at risk (OARs) to fractionated RT (3 to 5 fractions) are relatively limited and dose constraints remain not validated [20, 21]. Current clinical recommendations indicate that doses to brainstem, optic apparatus, and spine should not exceed a maximum point dose of 23 Gy, 19.5 Gy, and 22.5 Gy when using 3 fractions, and 31 Gy, 25 Gy, and 30 Gy when using 5 fractions, respectively.

Different factors may alter the risk of radionecrosis following reirradiation for brain tumors, including dose and fractionation, target volumes, combined systemic treatments, and interval between the RT treatment courses [22, 23]. In their meta-analysis of 30 studies published from 1996 to 2011, Sminia and Mayer [22] found no cases of radionecrosis for a cumulative biologically effective dose normalized to 2 Gy/fraction (EQD2) of < 96 Gy using the linear quadratic model and assuming an α/β ratio of 2 Gy for normal health brain. The median cumulative EQD2 was generally higher in SRS series (111.6 to 137.2 Gy) than in hypofractionated (90 to 133.9 Gy) and conventionally fractionated reirradiation (81.6 to 101.9 Gy) series. For patients with recurrent glioblastoma receiving hypofractionated SRT or SRS, brain necrosis was reported in 2–12% for a cumulative EQD2 > 96.2 Gy and up to 17% of for a cumulative EQD2 > 137 Gy. In recent update of the literature on reirradiation of glioblastoma, a similar risk of radionecrosis of 0 to 3% has been shown after conventional fractionation with cumulative EQD2 doses < 101 Gy, of 7 to 13% after hypofractionated SRT with cumulative EQD2 doses of 102 to 130 Gy, and up to 24.4% after SRS with cumulative EQD2 doses of 120 to 150 Gy [23].

The relationship between cumulative EQD2 values and risk of toxicity for sensitive brain structures has been evaluated in few retrospective studies [24, 25]. Niyazi et al. [24] found no relevant long-term toxicity in a series of 58 patients who received reirradiation for a malignant glioma using maximum EQD2 values of 80.3 Gy, 79.4 and 95.2 Gy to the optic chiasm, optic nerves and brainstem, respectively, considering an α/β ratio of 2 Gy for these structures. In a systematic review on reirradiation of diffuse brainstem gliomas including seven studies with a total of 90 patients, Lu et al. [25] showed that a second course of radiation was associated with clinical improvement and radiological response without significant toxicity employing doses of 20–24 Gy given in 2 Gy fractions. Overall, these data indicate relatively high and fast recovery capacity of the normal human brain after RT like those seen for spine [4, 5], and support the relative safety of reirradiation using cumulative EQD2 doses around 100 Gy, or even higher (up to 120 Gy) for small and well-defined recurrent tumors away from eloquent areas.

### Radiotherapy techniques and target delineation

High accuracy in tumor localization, target dose coverage and dose delivery are crucial when performing a second course of focal RT. In this setting, stereotactic techniques are frequently employed for their ability to achieve a steep dose fall-off at the edge of the target volume lowering the radiation dose to surrounding sensitive brain structures. Current stereotactic techniques include Gamma Knife (Elekta Instruments AB, Stockholm, Sweden) and linear accelerator (LINAC)-based SRS systems, such as CyberKnife (Accuray, Sunnyvale, CA, USA) or Novalis (NTx) (BrainLAB AG, Feldkirchen, Germany). Patients receiving Gamma Knife SRS are traditionally placed in a rigid stereotactic frame with a submillimetric target accuracy, while those treated with LINAC-based frameless SRS systems are usually immobilized using a thermoplastic mask. A submillimeter accuracy of patient positioning is achieved using modern image guided radiation therapy (IGRT) technologies, such as orthogonal x-rays (ExacTrac^®^Xray 6D system) or cone beam CT (CBCT) [2]. Radiation dose is usually delivered in a single fraction to targets smaller than 3 cm in size, while hypofractionated and conventionally fractionated schedules are frequently used for treating larger recurrent tumors. Highly conformal dose distribution can be achieved with IMRT and VMAT techniques. Currently, no comparative studies have demonstrated the clinical superiority of a technique over another in patients with brain tumors in terms of local control and treatment-related toxicity. In proton therapy, there are two main techniques of irradiation, namely active scanning or pencil beam scanning and passive scattering proton therapy. Limited data suggest that proton therapy is an effective treatment for recurrent brain tumors [26, 27], although there are no controlled studies demonstrating its superiority in comparison to photon RT in terms of local control and decreased toxicity.

An accurate delineation of tumor volumes and OARs is essential for a precise calculation of the spatial dose distribution and for the optimal radiation schedule. For brain tumors, the gross tumor volume (GTV) is generally defined as the visible lesion on MRI contrast-enhanced T1-weighted sequences. The clinical target volume (CTV), which includes areas of potential suspected microscopic tumor infiltration and potential paths of microscopic spread, can be generated by adding a variable margin of up to 5 mm to the GTV constrained at anatomical borders, e.g. tentorium, falx cerebri, and bone. In general, little (1–2 mm) or no GTV-to-CTV margins are used during SRS with the aim to limit the risk of toxicity, where larger margins up to 5 mm are commonly applied during hypofractionated and conventionally fractionated SRT [23]. Advanced MRI techniques, e.g. diffusion MRI and perfusion MRI, and positron emission tomotherapy (PET)/CT imaging with radiolabeled amino acids may help to improve target volume delineation accuracy by revealing tumor infiltration, although their use in clinical practice are limited and more evidence need to confirm their usefulness [28]. Finally, depending on radiation technique and available technology, an expansion of 0 to 3 mm is applied to generate the planning target volume (PTV) which accounts for uncertainties in treatment planning and patient positioning. Whole brain radiation therapy (WBRT) and craniospinal irradiation (CSI) can be used for selected patients with recurrent tumors that have spread into the brain and spinal cord through the cerebrospinal fluid, e.g. ependymoma and medulloblastoma.

### Glioblastoma

Reirradiation is increasingly used as treatment option in patients with recurrent glioblastoma after standard chemoradiation [29–59]. A summary of selected reirradiation studies reporting survival and toxicity rates after different radiation schedules with or without systemic therapies is shown in Table 1. Median survival times from 7 to 13 months and 1-year OS rates of 30–55% have been observed following either SRS or fractionated SRT, with 1-year incidence of neurological toxicities ranging from 5 to 20%. Minniti et al. [23] reported the clinical outcomes of 901 patients treated with single-fraction SRS for recurrent glioblastoma included in 16 studies published between 2005 and 2020. Using a median dose of 15–18 Gy, median OS ranged from 7.5 to 13 months and median PFS from 4.4 to 6 months. Even though Gamma Knife was the most used SRS technique, clinical results were no different for patients treated with Cyberknife or LINAC-based SRS. In a recent systematic review of reirradiation with different SRS modalities for recurrent glioblastoma including 50 studies (2096 patients), Kazmi et al. [60] observed similar 12-month OS and PFS rates of 34% and 16%, respectively.

**Table 1:** Selected studies of reirradiation for recurrent glioblastoma

Author	No pts	RT Type	Median Dose (Gy/fr)	Concurrent Systemic Therapy (N)	Interval between RT courses (months)	Median PFS (months)	Median OS (months)	RN (%)
Combs et al., 2005	59	FSRT	36/18	TMZ or PVC, (36)	10	5	8, 23% at 12 months	0
Grosu et al., 2005	34	HSRT	30/6	TMZ (29)	16	NR	8 (both), 11 (RT + TMZ), 6 (RT alone)	20.5
Kong et al., 2008	65	SRS	16/1	None	4.3	4.6	23	37.5
Cuneo et al., 2009	49	SRS	15/1	BEV	20	5.2 (+ BEV), 2.1 (-BEV)	11.9 (+ BEV), 3(-BEV)	10
Gutin et al., 2009	20	HSRT	30/5	BEV	15	7.3 (4.4–8.9)	12.5; 54% at 12 months	0
Fogh et al., 2010	105	HSRT	35/10	TMZ (26), other (22)	8	NR	11	0.7
Minniti et al., 2011	36	HSRT	37.5/15	TMZ	14	5; 42% at 6 months	9.7; 33% at 12 months	22.2
Minniti et al., 2013	38	HSRT	30/5	TMZ	15.5	6 24% at 12 months	12.4; 53% at 12 months	
Martinez-Carrillo et al., 2014	46	SRS	18/1	NR	10	NR	7.5	10
Wick et al., 2014	91	FSRT	36/18	APG101 (58)	21	2.5 (RT), 4.5 (RT + APG101)	11.5 (both groups)	1.3
Kim H.R. et al., 2015	57	SRS	15/1	TMZ	8.8	3.6 (2.3 + TMZ)	9.2 (15.5 + TMZ)	NR
Minniti et al., 2015	42	HSRT	25/5	FTM (23) BEV (19)	14	50% (BEV), 18% (BEV + FTM) at 6 months	30% (BEV), 8.3% (BEV + FTM) at 12 months	16.6
Pinzi et al., 2015	88	SRS	16–22/1	NR (22)	15	NR	11.5 48% at 12 months	6
Imber et al., 2017	174	SRS	16/1	TMZ (20), CCNU (13), BCNU (11)	8.7	NR	10.6	13
Kim et al., 2017	57	SRS	15/1	TMZ (28)	NR	3.6, 6 (+ TMZ)	9.2, 15.5 (+ TMZ)	24.4
Sharma et al., 2017	53	SRS	18/1	None	16	4.4	11	4
Palmer et al., 2018	87	SRT	35/10	none	10.8	NR	13.9	NR
Fleischmann et al., 2019	124	FSRT	36/18	BEV (95)	18	5	9	6.9
Scartoni et al., 2020	33	PBRT	36/18	TMZ (7)	21.3	5.9	8.7	9.09
Kaul et al., 2020	133	HSRT	41.8–49.4/12–15	TMZ (58)	14	NR	6	5.6
Saeed et al., 2020	45	PBRT	42.6/20	TMZ (16), BEV (4), TMZ + BEV (10)	20.2	13.9	14.2	8.8
Attia et al., 2022	57	FSRT	36/18	none	16	8	11	3.5
Tsien et al., 2023	170	HSRT	35/10	BEV + RT, BEV alone	NR	54% vs. 29% at 6 months	10.1 BEV + RT, 9.7 BEV alone	0

Legend: BEV, bevacizumab; BCNU, Carmustina; CCNU, Lomustine; FSRT, fractionated stereotactic radiotherapy; FTM, fotemustine; HSRT, hypofractionated stereotactic radiotherapy; NR, not reported; OS, overall survival; PBRT, proton beam radiotherapy; PFS, progression-free survival; PVC, Procarbazine, lomustine, vincristine; RN, radionecrosis; SRS, stereotactic radiosurgery; SRT, stereotactic radiotherapy; TMZ temozolomide

Hypofractionated SRT, given as moderate hypofractionation (35-37.5 Gy in 10–15 fractions of 2.5–3.5 Gy each) or as high-dose hypofractionation (27–35 Gy in 3–5 fractions of 5–9 Gy each) is increasingly used in the setting of reirradiation as an alternative to single-fraction SRS (Table 1) [31, 34–39, 43, 50, 53–55, 59]. Fogh et al. [36] observed a median OS of 11 months in 105 patients with relapsed glioblastoma who received a total dose of 35 Gy in 10 fractions. In a recent review reporting the outcome of hypofractionated SRT for 995 patients with recurrent glioblastoma included in 17 studies, a similar median OS time of 9.2 months (ranging from 7.5 to 12.5 months) has been observed in patients treated with SRT using doses of 30–45 Gy in 2.5-4.0 Gy fractions and those receiving 25–35 Gy in 5–7 Gy fractions [23].

A similar OS of 7 to10 months has been observed using conventionally fractionated SRT in 2 Gy fractions [30, 41, 51, 58]. In 172 patients with recurrent low- and high-grade gliomas who were treated with 36 Gy delivered in 2-Gy fractions, Combs et al. [30] observed median OS and PFS times of 8 and 5 months, respectively. Histology significantly influenced outcomes. Median OS was 8 months for patients with GBM, 16 months for patients with grade 3 tumors, and 22 months for patients with low-grade gliomas; respective median PFS times were 5, 8, and 12 months.

Symptomatic brain necrosis is a serious late consequence of reirradiation, with an incidence ranging from 0 to 24.4% at 1 year (Table 1). The reported risk of radionecrosis is < 10% for cumulative EQD2 < 100–110 Gy and rises to 25% for cumulative EQD2 > 130 Gy using an α/β ratio of 2 Gy for normal health brain. Considering an EQD2 of 60 Gy for the initial standard chemoradiation, this means in clinical practice that reirradiation doses of 15–16 Gy given as single fraction (EQD2 = 63.7–72 Gy) or 25 to 30 Gy delivered in 5 fractions (EQD2 = 43.7–60 Gy) carry an acceptable risk of radionecrosis around 10%, at least for patients with relatively small tumor volumes. A low risk ranging from 0.8 to 6.8% has been observed following reirradiation using conventionally fractionated SRT with a median total dose of 36 Gy in 2-Gy fractions (EQD2 = 36 Gy), even in patients with large target volume around 100 ml or higher, or when using large safety GTV-to-CTV margins up to 10 mm.

Superior survival benefit of reirradiation in combination with systemic therapy remains matter of debate. In a few retrospective studies, the combination of RT with alkylating agents offered longer OS and PFS times compared with RT alone, but this benefit seems to be limited to MGMT methylated tumors [31, 37–39]. In contrast, other few series failed to show significant survival benefit with the addition of chemotherapy to RT [36, 53, 61]. Retrospective studies observed significantly longer OS with the addition of bevacizumab to SRS and SRT compared to reirradiation alone [33, 48, 51, 62]. Another controversial issue is the potential superiority of combining systemic therapy with reirradiation versus systemic treatment alone [59, 63]. In the secondary analysis of NRG Oncology/RTOG trial 0525 evaluating dose-dense versus standard dose temozolomide in newly diagnosed glioblastoma, Shi et al. [63] investigated the impact of different salvage treatments in 637 patients with recurrent or progressive GBM. Median survival times were 12.2, 8.2, 10.6, 4.8 months, respectively, in patients receiving bevacizumab (44%), reirradiation alone (4%), combined radiation and systemic therapy (10%), or no treatment (42%). Although patients receiving no salvage treatment had significantly lower survival than the others, survival analysis failed to show significant differences among patient groups who received bevacizumab with or without reirradiation. In the NRG Oncology/RTOG 1205 phase II randomized trial of 182 patients with recurrent glioblastoma who received hypofractionated SRT (35 Gy in 3.5 Gy fractions) and concurrent bevacizumab or bevacizumab alone, Tsien et al. [59] observed similar median survival times of 10.1 and 9.7 between groups; however, the combined treatment was associated with better 6-month PFS (54% versus 29%, p < 0.001). The treatment was well tolerated with few (5%) acute and no delayed grade ≥ 3 toxicity, confirming the safety of reirradiation with modern RT techniques.

In summary, reirradiation is a feasible treatment option in selected patients with recurrent diffuse gliomas. An appropriate patient selection is essential to achieve survival benefit. According to international recommendations and prognostic score indexes, reirradiation should be considered in young patients with good performance status, and at least 6 months interval from the first course of RT [61, 64–68]. Survival benefit is longer in patients with lower grade gliomas compared with glioblastoma. Choosing the appropriate radiation technique according to tumor size and location is a key factor in the management of these patients to achieve better clinical outcomes while limiting the potential toxicity. SRS given in one or few fractions can be recommended for small to moderate targets up to 3-3.5 cm in size, while fractionated SRT using doses of 1.8 to 3.5 Gy per fraction should be preferred for larger tumors, especially those close to eloquent structures. Although the combination of reirradiation and bevacizumab did not significantly improve OS for patients with recurrent glioblastoma in NRG Oncology/RTOG1205, the meaningful improvement in the 6-month PFS rate with combined treatment remains an important goal which is clinically beneficial in this disease with limited treatment options. The potential superiority of combining a second course of RT with alkylating agent lomustine (the standard systemic treatment for recurrent glioblastoma in Europe) over lomustine alone will be evaluated in a prospective randomized EORTC phase III trial (LEGATO trial) which will start enrolling patients in Q1 2024 in Europe.

### Ependymoma

Ependymomas are rare CNS tumors of neuroectodermal origin that can affect both the pediatric and adult populations, with about 15% of all patients being children of less than 5 years of age [69]. Maximal safe resection followed by adjuvant RT to the tumor bed represents the standard of care [70]. Recurrent disease may occur in 30–50% of patients and it is treated by local excision plus reirradiation as systemic therapies have proven to be of a little benefit. Reirradiation given as focal treatment or CSI RT has been associated with survival benefit [71–79] (Table 2).

**Table 2:** Selected studies on reirradiation for recurrent ependymomas

Author	No pts	RT modality	Median RT dose at recurrence (Gy/fr)	Median upfront RT dose (Gy/fr)	interval between RT courses (months)	Median OS (months)	Median PFS (months)	RN
		(No patients)						(%)
Hoffman et al., 2014	12	HSRT	24/3	55.8–59.4/30	25	71% at 2years	32 s, 89% control at 3 years	50 (25 symptomatic)
Lobón et al., 2016	32	FSRT (24) HSRT (8)	54/30	54/30	14	42	14 (0.7 years after FSRT; 6.8 years after CSI)	15.6
			35/10					
Tsang et al., 2018	101	FSRT (46) FSRT + CSI (55)	54/30; 39.6/22 (CSI)	59.4	26.8	75.1: 57.3% at 5 years	26.7: 37.3% at 5 years	7 (7.9 at 10 years)
Tsang et al., 2019	31	FSRT (15) FSRT + CSI (16)	54/30; 39.6/22 (CSI)	54-59.4	23	53.1; 3 and 5-year 62.8% and 39.9%	23.3; 3 and 5-year OS 38.6% and 24.1%	9
Régnier et al. 2019	31	SRS (4) HSRT (18) FSRT (11)	18/1; 44/10; 54/30	57.6	37	34	31 (median local recurrence-free survival)	3
Gupta et al., 2020	55	FSRT	54/30	55.8	37	40% at 3 years	51% at 3 years	12.5 (5.5 symptomatic)
Mak et al., 2021	35	FSRT (26) CSI + FSRT (7) SRS (2)	54/30; 44/10 15–24/1	55.8	67	65; 56.9 after FSRT and not reached after FSRT + CSI	33; 23.1 after FSRT and not reached after FSRT + CSI	3

Legend: CSI, Craniospinal irradiation; FSRT, fractionated stereotactic radiotherapy; HSRT, Hypofractionated stereotactic

radiotherapy; OS, overall survival; PFS, progression-free survival; Pts, patients; RN, radionecrosis; RT2, second course of

radiation therapy; SRS, stereotactic radiosurgery

Tsang et al. (2018) [75] evaluated 101 patients with recurrent ependymoma treated with a second course of fractionated RT after prior focal RT given to a dose of 54 Gy in 1.8 Gy daily fractions. Recurrent tumors received a median dose of 39.6 Gy delivered in 1.8 Gy daily fractions to sites of gross or resected recurrent tumor using either photons (n = 88) or protons (n = 13); 55 patients with recurrent ependymoma were treated with CSI. With a median interval of 26.8 months between the two courses of RT, median durations of OS and freedom from progression were 75.1 and 27.3 months, respectively; and 2-year OS and freedom from progression rates were 74.9% and 53.3%, respectively. CSI was associated with improved outcome, whereas male sex, anaplastic histology at recurrence, treatment group, and a short interval between RT courses were associated with a worse outcome. Grade 1–3 radiation necrosis occurred in 25 patients, with a 10-year cumulative incidence of 26.9%, being of grade ≥ 3 in seven patients (7.9% at 10 years). A similar local control was reported in other retrospective series using both single-fraction SRS (15–24 Gy) or three-fractions SRS (7–8 Gy per fraction), although with an increased risk of radionecrosis up to 50% compared with conventionally fractionated schedules [72, 79, 80].

The question whether CSI as part of reirradiation could improve the clinical outcome compared with focal reirradiation has been addressed in a retrospective study conducted at the Hospital for Sick Children and Princess Margaret Cancer Center in Toronto between 1999 and 2018 [79]. Patients with locally recurrent ependymoma treated before 2012 received focal reirradiation whereas those treated from 2012 received CSI, 23.4–36 Gy in 1.8 Gy daily fractions, followed by boost to the site of resected/macroscopic disease. Among 22 patients with local failure after the first course of RT, the use of CSI as reirradiation was associated with significant improvement in time to recurrence; median time and 5-year rate of time to recurrence were 26.7 months and 15.2% in those who did not receive CSI, respectively, versus not reached and 83.3% for those who received CSI (p = 0.03). However, this difference did not translate into a statistically significant OS difference maybe because of the small number of patients. The treatment was safe, with only one patient who developed grade 3 radionecrosis.

In summary, reirradiation of ependymoma represents an effective treatment approach in patients with locally recurrent lesions after failure of previous adjuvant focal irradiation. The use of single-fraction SRS has been associated with an increased risk of adverse effects compared with conventionally fractionated schedules, especially for larger intact or resected tumors. Preliminary data suggest that CSI as a component of reirradiation offers a statistically significant PFS benefit compared with focal reirradiation, although large series with long-term follow-up are needed to confirm its survival benefit. There is published evidence supporting the use of proton beam therapy for its potential ability of reducing late toxicity in patients receiving CSI [81–84]. In this regard, results of a prospective study of surgery and fractionated re-irradiation with photon or proton RT in patients for recurrent ependymoma are expected in 2028 (ClinicalTrials.gov, number NCT02125786).

### Diffuse midline gliomas

Diffuse midline gliomas H3 K27 altered (previously called diffuse intrinsic pontine gliomas - DIPGs) are extremely aggressive WHO grade IV tumors and represents a leading cause of brain tumor deaths in children, with 90% of children dying within 2 years from the initial diagnosis. According to the Fifth WHO Classification published in 2021 [85], diffuse midline gliomas are characterized by diffuse infiltrative growth in the brain tissue, involvement of midline structures (thalamus, brain stem and spinal cord) and harbor H3 K27M-mutation. RT, using 54–60 Gy in 1.8-2.0 Gy fractions remains the standard of care, but its role is mainly palliative and provides only temporary relief [86, 87].

Few studies investigated clinical outcomes of patients with recurrent/progressive diffuse midline glioma treated with reirradiation [88–93]. Using median doses for reirradiation of 18–24 Gy in 1.8-2.0 Gy daily fractions, median OS reported in six published studies ranges from 4 to 8.3 months and median PFS from 3 to 4.5 months from the time of reirradiation (Table 3). A retrospective European study has evaluated benefit and toxicity of reirradiation in 31 patients with diffuse midline glioma at first progression [89]. Most patients were treated with a conventionally fractionated regimen up to a total dose of 20 Gy in 1.8-2.0 Gy daily fractions, given alone or in combination with systemic therapy. Following reirradiation, the reported median survival time was 6.4 months compared to 3 months in a historical cohort of 39 patients receiving no treatment at time of progression (median survival of 13.7 versus 10.3 months after upfront RT). In addition, a clinical improvement was noted in nearly 80% of the patients with no life-threatening or fatal toxicities observed during the follow-up. Longer interval between RT courses was an independent factor for longer survival; in contrast, the addition of systemic therapy and age did not influence survival. In another Canadian retrospective study including 14 patients with diffuse midline glioma who received focal reirradiation using doses of 21.6 to 36 Gy given in 1.8 Gy daily fractions, median OS from reirradiation was 6.5 months compared to 3 months in historical cohorts of 46 patients not treated with reirradiation [91]. Similar OS benefit of a second course of fractionated RT have been confirmed in other few small retrospective series [92, 93].

**Table 3:** Selected studies on reirradiation for recurrent Diffuse Midline Gliomas

Author	No pts	RT modality (No)	Median Dose (Gy/fr)	Concurrent systemic therapy (No)	Median interval between RT courses (months)	Median PFS (months)	Median OS (months)	Grade 3 RN (%)
Massimino et al., 2014	11	cFRT	19.8/11	none	NR	8.3	6	0
Janssens et al., 2017	31	cFRT	20/10	yes (e.g. TMZ; Nitozumab + vinorelbine) (15)	Minimum 3 months	8.2	6.1 (RT + CHT) 5.4 (RT)	0
Kline et al., 2018	12	cFRT HFRT	24/12; 24/10	Nivolumab (8)	11.6 (RT + N) 12.1 (RT alone)	4.2 (RT + N) 4.1 (RT)	6.8 (RT + N) 6 (RT)	0
Lassaletta et al., 2018	16	cFRT	30.6/17	BEV (1)	13 (from diagnosis)	4.5	6.5	6.2
Amsbaugh et al., 2019	12	cFRT HFRT	24/12 (6 pts); 26.4/12 (4 pts); 30.8/14 (2 pts)	none	12.8	4.5	5.8	0
Krishnatry et al., 2021	20	cFRT	39.6–45/22–25	none	8.9	NR	5.5	0

Legend: BEV, bevacizumab; cFRT, conventionally fractionated radiotherapy; CHT, chemotherapy; HFRT, hypofractionated radiotherapy; N, nivolumab; NR, not reported; OS, overall survival; PFS, progression-free survival; Pts,patients. RN, radionecrosis

No severe neurotoxicity related to reirradiation has been observed using total doses < 24 Gy (1.8-2.0 Gy per fraction) [90–92]. Amsbaugh et al. [92] evaluated imaging changes, clinical symptoms, and patient- or family-reported quality of life in a prospective phase I/II trial of diffuse midline glioma receiving reirradiation at the time of tumor progression. From the start of reirradiation, median PFS and OS time were 4.5 and 5.8 months, respectively. Six patients who received 24 Gy in 12 fractions and 2 out of 4 patients who received 26.4 Gy in 12 fractions demonstrated improvement in clinical symptoms and quality of life without grade 3 toxicity. In 2 patients who received 30.4 Gy in 14 fractions, grade 3 toxicity occurred in one patient.

In summary, a second course of RT can be considered in children with DPG H3 K27 altered. A few studies demonstrated a median survival of 5 to 7 months following reirradiation, although all data come from retrospective series. Clinical benefit can be observed in up to 80% of patients and this has been associated with an improvement in quality of life. Severe toxicities from reirradiation appear to be limited using conventionally fractionated RT schedules with doses of 20 to 24 Gy. Regarding the timing of reirradiation, interval of at least 6 months between the two radiation treatments is associated with better outcome. Future clinical trials need to assess optimal dose, fractionation, interval between treatments, and the concurrent use of systemic agents in such patients.

### Brain metastases

SRS is the recommended treatment for patients with a limited number of brain metastases (1–4 lesions), resulting in a significant decrease of neurocognitive decline compared to WBRT without detrimental effects on OS [94, 95]. In the recent ESMO-EANO and ASTRO guidelines on treatment of brain metastases [96, 97], SRS has been also recommended for patients with 5–10 lesions. The reported local control following SRS is around 75 to 90% at one year, with late local recurrences that are increasingly observed. For patients with locally recurrent brain metastases, repeat SRS is a challenging treatment because of the difficulty of discerning progression from treatment effect and the increased risk of radionecrosis. A summary of selected reirradiation studies for brain metastases is shown in Table 4 [98–105]. With a variable median follow-up of 7–19 months, local control ranges between 70% and 95% at 1 year and the risk of symptomatic radionecrosis is around 7–16% for 546 patients included in eight selected studies (Table 4).

**Table 4:** Selected studies on reirradiation for recurrent Brain Metastasis

Author	No Pts	Most common tumor histology (No)	SRS dose Gy/fr	Concurrent Systemic Therapy	Interval between SRS courses (months)	Median follow-up (months)	Median OS (months)	Median LC rate	Symptomatic RN (%)
Terakedis et al., 2014	37	Melanoma (20), Lung (9), Breast (8)	18/1	NR	9	7	8.3	80.6% at 1 year	16; 16.5 at 12 months
Minniti et al., 2015	43	NSCLC, Breast, Melanoma, Others	21–24/3	none	17	19	10	70% 1 year	14 grade 2 or more); 34 at 12 months
Shultz et al., 2015	95	Melanoma (16), Lung (38), Breast (14), GI (11)	22/1 (91%) 24/3 (8%)	NR	4	15	11	95% at 1 year	7.6
Balermpas et al., 2018	31	Breast (10), NSCLC (10), Melanoma (5), Other (6)	1 fr (24) 3–5 fr (7)	Concurrent targeted therapy (14)	12.4	11.9	61.7% and, 46.3% at 1 and 2 years	79.5% and 71.5% at 1 and 2 years	12.9, grade 3 or more
Jiang et al., 2019	63	Lung (45), Breast (8), Colorectal (3), Renal (2), Other (5)	20/1 30/2	none	10	12	18	94.4% at 1 year	14.2
Kowalchuk et al., 2021	102	NSCLC	18/1	none	12	14	NR	79% and 72% at 1 and 2 years	7
Bhatia et al., 2022	51	NSCLC (19), Breast (12), Other (20)	24/3	none	NR	32	14.1	79.5% at 1 year	10.9
Sneed et al., 2022	124	Breast, Lung, Melanoma	18/1	TKI, ICI	15.4	13.4	18.6	80% at 1 year	10

Legend: GI, Gastrointestinal; ICI, immune checkpoint inhibitors; LC, local control; NSCLC, non-small-cell lung cancer

NR, not reported; OS, overall survival; PFS, progression-free survival; Pts, patients; RN, radionecrosis;

SRS, radiosurgery; TKI, tyrosine kinase inhibitors

Sneed et al. [105] evaluated the efficacy and safety of repeat single-fraction SRS in terms of treatment failure and risk of adverse radiation effects for 124 patients with 229 recurrent brain metastases from various cancer types, the most common from breast cancer, lung cancer, and melanoma. With a median SRS prescription dose of 18 Gy and median follow-up of 14.5 months, the 1-year freedom from progression was 82% and risk of symptomatic adverse radiation effects 11% for lesions with a quadratic mean diameter of 0.75-2.0 cm. For lesions with a quadratic mean diameter of 2.01-3.0 cm, SRS was associated with 1-year control rates of 65% and a higher risk of symptomatic adverse radiation effects of 24%. In another multi-institutional retrospective series of 102 patients with 123 brain metastases treated with repeat SRS after local or marginal recurrence after prior SRS, Kowalchuk et al. [103] reported 1-year local control rates of 79% and 1-year incidence rates of symptomatic adverse radiation effects of 7%. Tumor control was significantly better for lesion ≤1 cm (p < 0.005). The risk of symptomatic radionecrosis was higher for cumulative maximum doses ≥ 40 Gy or for a volume of normal brain receiving 12 Gy > 9 cm^3^ at the time of repeat SRS (p < 0.025). Similar 1-year local control rates of 70–80% and symptomatic radionecrosis rates around 7–16% have been shown in other published series of repeat SRS after either prior SRS or WBRT [98, 101].

Fractionated SRS (2–5 fractions) has been suggested as an alternative treatment option to single-fraction SRS for locally recurrent brain metastases [99, 100, 104, 106]. In a systematic review and meta-analysis of stereotactic reirradiation for local failure of brain metastases following previous SRS, Loi et al. [106] reported clinical outcomes for 335 patients with 389 brain metastases treated with either single-fraction (n = 282) or fractionated (n = 107) SRS. With a median follow-up of 12 months from the time of repeat SRS, median OS time was 14 months, 1-year local failure was 24%, and crude cumulative incidence of radiation necrosis was 13%. There were no differences in local control and risk of symptomatic adverse radiation effects with single-fraction SRS using doses of 16–19 Gy or fractionated SRS using a total dose of 21–24 Gy given in 3 fractions, although the median volume of lesions receiving 3-fractions SRS was generally larger. No factors were associated with an increased risk of radionecrosis, including tumor volume, histology, higher biological effective dose, and longer time interval from first SRS.

Overall, repeat SRS has emerged as an effective strategy for patients with recurrent brain metastases. In the respect of the relatively small number of patients reported and retrospective nature of studies, either single-fraction or fractionated SRS are associated with high local control and acceptable risk of symptomatic radionecrosis < 15%. Optimal radiation dose and fractionation for different target volumes, as well safe combination with systemic agents need to be defined.

### Other brain tumors

Reirradiation has been used as possible salvage treatment option for several other recurrent brain tumors. In patients with recurrent medulloblastoma after standard CSI and a boost to the posterior fossa/tumor bed, small retrospective studies observed survival rates of 50–75% at 1 year following a second course of RT, with higher rates for those presenting with focal recurrences versus diffuse leptomeningeal disease [107–111]. Hypofractionated schedules (25–30 Gy in 3 to 10 fractions) are typically used for focal radiation while a dose of 20–24 Gy in 1.8 Gy daily fractions is used to the entire spine. Single site of recurrence, minimal residual disease, time from the first course of RT, and molecular subtypes are known to affect survival [112]. The risk of toxicity is low when cumulative EQD2 for brain and spine does not exceed 150 Gy and 120 Gy [7, 8]. Few published series suggest that a second course of RT, both SRS and SRT using either photons or protons, may be a feasible salvage treatment option for selected patients with skull base recurrent tumors, including recurrent aggressive pituitary adenomas [113, 114] and meningiomas [115–117], which is associated with a risk of symptomatic radionecrosis, cranial deficits, and radiation-induced optic neuropathy < 15%.

## Conclusions

This review represents a synthesis of the available literature data and a basis for further consideration. An increasing number of studies indicates that reirradiation is a feasible treatment option among patients with recurrent brain tumors. Although caution is required when performing a second course or RT for the increased risk of radiation-induced toxicity, most studies using modern radiation techniques indicate that retreatment is associated with a risk of adverse radiation effects < 10% for cumulative EQD2 doses of 100 to 110 Gy in patients with recurrent brain lesion and for cumulative EQD2 doses of 70 to 75 Gy in those with recurrent spine lesions assuming an α/β ratio of 2 Gy for normal tissue. While available data support the use of reirradiation as salvage therapy in selected patients with brain tumors, a definitive judgment on the efficacy and safety of a second course of RT and its superiority over other treatment options (systemic treatment or repeat surgery) cannot be made because of the small number of patients and the retrospective nature of most studies. Only prospective studies with appropriate follow-up can confirm OS benefit of reirradiation for different tumors, as well to address unanswered questions such as optimal radiation dose and fractionation, target volumes delineation, combination of reirradiation with new systemic agents and immunotherapy, and which patients will benefit most from treatment. Until these data become available, the decision to offer reirradiation in clinical practice to patients with recurrent tumors to improve disease control and OS should be always weighed against the potential toxicity of treatment.
